# How animal milk and plant-based alternatives diverge in terms of fatty acid, amino acid, and mineral composition

**DOI:** 10.1038/s41538-023-00227-w

**Published:** 2023-09-16

**Authors:** S. S. Moore, A. Costa, M. Pozza, T. Vamerali, G. Niero, S. Censi, M. De Marchi

**Affiliations:** 1 Department of Agronomy, Food, Natural Resources, Animals and Environment (DAFNAE), University of Padova, Padova, Italy; 2 Department of Veterinary Medical Sciences (DIMEVET), University of Bologna, Padova, Italy; 3 Department of Medicine, Endocrinology Unit, University of Padova, 35121 Padova, Italy

**Keywords:** Proteins, Agriculture, Nutrition

## Abstract

The decline in fresh milk in the Western world has in part been substituted by an increased consumption of plant-based beverages (PBB). These are often marketed as healthy and sustainable alternatives to milk and dairy foodstuff, although studies have suggested PBB to be of lower nutrient quality. The current study considered different brands of almond-, oat-, rice-, coconut- and soya-based beverages for a comparative analysis and found that they indeed presented lower contents of total protein, lipids, amino acids, and minerals than cow and goat milk. The only exception was given by soya-based beverages which approximated the protein content (3.47% vs. 3.42 and 3.25% in cow and goat milk, respectively) and amino acid composition of animal milk, and also demonstrated high mineral content. The natural presence of phyto-compounds in PBB characterised as antinutrients and their potential to exacerbate the issue of low nutrient quality by lowering bioavailability have been discussed.

## Introduction

Dairy products have been historically important sources of nutrients particularly in the Western world and in certain Asian countries. Today their consumption is recommended in numerous national dietary guidelines for their high levels of essential nutrients including minerals, fatty acids (FA), and proteins^1^. Indeed, dairy provides 49% of global dietary calcium, 15% of dietary fat, and 12% of proteins^2^. Despite this, the consumption of animal milk is in decline in Europe and the U.S. In 2011 the per capita fresh milk consumption in the EU amounted to 56.3 kg, but a 14.6% decline has been projected between 2011 and 2031^3^, translating into a decrease of 394 mL/year. In the U.S. the annual consumption per capita in 2021 was 62.31 kg for milk, 2.92 kg for butter, and 17.2 kg for cheese (ref. ^4^; https://www.clal.it/en/?section=tabs_consumi_procapite), but the milk intake has been declining by 830 mL/year since 1975^5^. This decline has in part been replaced by a greater consumption of processed derivatives as well as plant-based beverages (PBB). These PBB are often marketed as healthier substitutes and are frequently, although improperly, referred to as “milk”. In Europe, the misuse of the term “milk” has led to the addition of the legally binding definition for milk in the Common Organisation of Markets Regulation (EU) No 1308/2013^6^. The term “milk” has thereby been banned on PBB labelling since 2013 to prevent consumer misguidance.

The recent expansion of non-dairy alternatives has largely been driven by issues concerning lactose intolerance and milk protein allergies^7^. The growing prevalence of veganism, awareness of animal welfare, and the perceived idea of lower environmental impact and improved health has further encouraged the growth of PBB on the mainstream market^8^. The most popular PBB are of almond, oat, soya, cashew and coconut origin, or a mixture of these^9^, but the innovative nature of this market allows for continues expansion of new products. As such, the nutritional content of PBB vary markedly depending on their plant origin, fortification, and industrial processing. Beyond the quantity of the major nutrients, i.e. lipids, carbohydrates, and proteins, the fractional profiles also greatly differ between animal milk and PBB, to which evidence suggest PBB to have a poorer nutritional profile^10^. Among micronutrients, PBB have demonstrated particularly poor in the mean content of the mineral I^10^. Iodine is a rate-limiting element for the synthesis of thyroid hormones that play a central role in growth and neurological development, especially in children^11^. Indeed, I deficiency represents the first preventable cause of brain damage worldwide and its recommended intake vary during lifespan^12^. Iodine is obtained almost exclusively from diets constituting seafood and dairy products as the main dietary components^13,14^. Salt iodisation and I fortification of common house-hold food products have therefore become frequent practices to avoid deficiency^15^.

To address other common micronutrient deficiencies associated with vegan and vegetarian diets, and to thereby be considered as appropriate substitutes to bovine milk, many PBB are often fortified with other minerals beyond I as well as with vitamins, appealing to perceived consumer health benefits. However, despite the growing demand and capitalization of PBB, research into the nutritional aspects of these beverages remains limited, with the exception given by the culturally important soya, whose culinary use has been documented for centuries in many diverse countries. Such research, however, is commonly conducted based on the declared nutrition content on the packaging rather than on composition measurements of actual contents. Indeed, studies have shown nutrient composition declared on food packaging does not always align with actual contents^16,17^. As milk is, above all, a precious source of high-quality protein and minerals, and its substitution for PBB may thus promote deficiencies of these nutrients, the present study aimed to (i) quantify the gross composition and the amino acids, fatty acids, and minerals in different PBB and animal milk (Table 1), and (ii) carry out a comparative analysis to elucidate which PBB types approximate the nutritional profile of animal milk. The present study applied the detailed FA and amino acid composition as well as gross composition and mineral content of several PBB and milk to identify which nutritive components best discriminate between the beverage types.

**Table 1:** Overview of the commercial beverages available.

Type	Brand	N	List of product ingredients declared on packaging
Rice	I	2	Water, rice (14%), sunflower oil, sea salt
	II	1	Water, rice (15%), sunflower oil, sea salt
	III	2	Water, rice (17%), rice oil, salt
	IV	2	Water, rice (17%), rice oil, salt
	V	2	Water, rice (16%), rice oil, salt
	VI	2	Water, rice (16%), cold-pressed sunflower oil, sea salt
Oat	I	2	Water, oat (14%), sunflower oil, salt
	II	2	Water, oat (13%)
	III	2	Water, oat (12%), sunflower oil, salt
	IV	2	Water, oat (16%)
	V	2	Water, oat (10%), sea salt
	VI	2	Water, oat (11.9%), sunflower oil, agave fibre, sea salt
Soya	I	2	Water, soya (8%)
	II	2	Water, soya (13.5%), sea salt
	III	2	Water, hulled soybeans (5.6%), vegetal fibre (3%), sugar, xanthan gum, sea salt
	IV	2	Water, soybeans (7%), sea salt
	V	2	Water, hulled Italian soybean (8%), sea salt
	VI	2	Water, soya (13.5%), sea salt
Almond	I	2	Water, sugar, almond (8%), sucrose ester, aromas
	II	2	Water, sugar, almonds (11%), E473, L-ascorbic acid, aromas
	III	2	Water, sugarcane, cream of peeled and toasted almonds (2%), xanthan gum, salt, natural aromas
	IV	2	Water, sugar, almond (5%), sucrose ester, aromas
	V	2	Water, almond (3%), sunflower lectin, sea salt
	VI	2	Water, almonds (5%), sea salt
Coconut	I	2	Water, coconut juice (30%), E473, E407, E412, coconut aroma
	II	1	Water, coconut paste (4%), cane sugar, rice starch, natural aromas, sea salt
	III	2	Light coconut milk (40%), water, coconut juice from concentrate (23%), sea salt
	IV	2	Water, coconut milk (8%; 60% coconut corresponding to 4.8% in the final product, water, guar gum), rice starch, guar gum, xanthan gum, aroma
	V	2	Water, coconut paste (4%), cane sugar, rice starch, natural aromas, sea salt
	VI	2	Spring water, coconut water (20%), coconut milk (9.4%; water, coconut (2.5%)), natural coconut aroma, gellan gum, salt
Cow	I	2	Whole Cow milk
	II	2	Whole Cow milk
	III	2	Whole Cow milk
	IV	2	Whole Cow milk
Goat	I	2	Whole goat milk, sodium citrate
	II	2	Whole goat milk
	III	2	Whole goat milk
	IV	2	Whole goat milk

Beverage types and the brands sampled as well as the list of ingredients declared on the packaging.

## Results and discussion

### Protein content and amino acid profile

The coefficient of variation (CV) was in general large and varied among beverage types (Table 2). Soya beverages contained the highest content of protein (3.47%), followed thereafter by cow (3.42%) and goat milk (3.25%). Indeed, legumes statistically differed from the cereal group, which constituted rice- and oat-based beverages, in terms of total protein content (Table 3). The fruit and crop groups did not significantly differ in total protein content, although an advantage was found in fruit-based beverages. Considering the single amino acids, both types of animal milk had higher content per unit volume of all essential amino acids than the soya-based beverages, except for Phe (Table 4), while soya had higher contents of Arg, Ser, Gly, Asp, Ala, and Cys. As expected, the PBB groups significantly differed between them in regard to the single amino acids, except for the fruit and crop group which except for His, Met, Cys, and Try, were similar in amino acid content (Table 4). It is evident that milk protein content and amino acid profile is much less variable than in the individual PBB, with particularly cow milk showing a low CV (Table 2). For example, the CV for Cys ranged between 19 and 46% for PBB, but only between 7 and 10% for milk. This demonstrates a lack of standardisation across PBB production in comparison to milk, inferring potential shortcomings in consumer assurance and in meeting consumer expectations in terms of nutrient content.

**Table 2:** The coefficient of variation (%) of all investigated traits within each beverage type.

Trait	Almond	Coconut	Oat	Rice	Soya	Cow	Goat
*Gross composition*							
Dry matter	64	55	13	12	13	2	5
Total protein	47	38	37	43	15	3	17
Lipids	50	43	142	53	42	4	17
Ash	48	23	21	31	20	3	19
Carbohydrates	77	74	16	16	64	2	1
Lactose	0	0	0	0	0	4	19
Glucose	111	119	97	56	91	174	209
Fructose	109	115	127	67	146	139	94
*Amino acids*							
His	41	127	97	36	43	5	35
Arg	43	38	40	74	19	5	27
Ser	46	35	29	63	16	5	22
Gly	48	39	25	50	13	5	25
Asp	44	37	32	51	13	3	20
Glu	44	37	32	66	12	4	22
Thr^a^	47	25	38	50	13	5	19
Ala	46	37	38	82	12	4	18
Pro	46	30	38	113	14	4	24
Lys^a^	58	45	48	69	15	5	20
Met^a^	77	47	58	130	39	21	30
Tyr	44	25	38	151	21	10	22
Val^a^	43	33	43	94	14	4	21
Cys	46	35	26	29	19	7	10
Ile^a^	42	255	56	53	14	4	20
Leu^a^	45	36	37	73	13	4	21
Phe^a^	97	62	55	90	78	73	186
Trp^a^	45	41	32	71	33	6	16
*Fatty acids*							
C4:0	0	332	0	0	0	19	10
C5:0	0	0	0	332	0	21	31
C6:0	0	35	243	292	199	11	9
C7:0	0	0	346	332	0	21	12
C8:0	197	34	303	280	245	7	9
C9:0	0	0	0	0	0	15	10
C10:0	252	33	328	154	249	5	7
C11:0	0	65	0	0	0	18	10
C12:0	222	34	339	141	346	5	8
C12:1	0	0	0	0	0	6	15
C13:0	0	51	346	0	0	12	17
C13:1	0	0	0	0	0	18	34
C14.0	83	34	280	61	46	4	5
C14.1	0	0	0	0	0	107	107
C15.0 iso	0	0	346	281	0	8	12
C15.0 anti	0	0	0	0	0	6	12
C15.0	0	0	273	79	346	107	109
C16.0	13	23	23	61	27	3	9
C16:1n7	77	206	89	33	75	104	79
C16:1n9	142	223	138	50	196	87	60
C17.0	108	229	68	38	20	4	20
C17.1	0	0	0	0	0	20	36
C18.0	14	40	73	53	28	6	10
C18:1n7	9	146	28	16	35	40	13
C18:1n9	11	145	30	35	38	7	5
C18:2n6^bd^	32	163	42	75	33	19	21
C18:3n3^bc^	175	206	67	88	29	42	31
C18:3n6^d^ & C19:0	0	0	0	332	0	84	84
C19:1	148	332	171	70	139	60	95
C20:0	56	27	59	69	29	27	14
C20:1	346	236	171	129	136	34	71
C20:1n9 & C20:1n7	136	145	73	62	63	0	283
C20:2n6^d^	0	0	182	223	221	283	0
C20:3n6^d^ & C21	0	0	0	0	346	15	121
C20.5n3^c^	346	332	95	332	0	112	114
C20:4n6^d^	0	0	0	0	0	29	18
C22:0	110	191	127	64	50	44	66
C22:1n9	167	332	67	144	0	0	283
C22:5n3^c^	0	0	0	0	346	110	86
C22:6n3^c^	0	0	210	332	0	0	0
C24:0	0	332	87	40	127	0	0
SFA	12	29	84	52	27	2	3
MUFA	11	145	30	33	37	4	6
PUFA	32	164	41	73	25	10	20
USFA	1	149	26	12	6	4	7
SCFA	0	332	0	0	0	19	10
MCFA	212	34	292	265	177	7	8
LCFA	0	5	4	2	0	1	2
VLCFA	82	114	56	49	37	20	26
*Minerals*							
I	236	0	0	0	0	49	73
Ca	39	30	42	43	21	7	30
P	46	34	30	29	23	8	28
Mg	322	190	128	173	32	11	21
K	48	49	18	63	29	6	7
Na	42	17	39	48	46	9	13
S	236	0	128	332	23	12	23

*SFA* saturated fatty acid, *MUFA* monounsaturated fatty acid, *PUFA* polyunsaturated fatty acid, *USFA* unsaturated fatty, *SCFA* short chain fatty acids, *MCFA* medium chain fatty acid, *LCFA* long chain fatty, *VLCFA* very long chain fatty,

^a^Essential amino acid.

^b^Essential fatty acid.

^c^Omega 3 family.

^d^Omega 6 family.

**Table 3:** Gross composition across beverages.

Trait	Rice	Soya	Coconut	Oat	Almond	Cow	Goat	Plant vs. animal	Fruit vs. crop	Monocot vs. dicot	Cereal vs. legume
Dry matter	12.31	7.78	4.14	10.02	6.60	12.22	11.84	***	**	*	***
Total protein	0.12	3.47	0.23	0.69	0.85	3.42	3.25	***	ns	***	***
Lipids	0.39	1.60	1.73	0.37	1.99	3.55	3.72	**	***	**	**
Ash	0.09	0.45	0.19	0.18	0.16	0.72	0.82	***	ns	***	***
Carbohydrates	12.6	1.55	1.95	8.00	8.20	4.90	4.35	ns	ns	^†^	***
Lactose	0.00	0.00	0.00	0.00	0.00	4.72	4.31	***	na	na	na
Glucose	3.12	0.33	0.37	1.04	0.97	0.01	0.00	***	ns	*	**
Fructose	0.06	0.33	0.26	0.06	1.02	0.02	0.02	***	**	***	***

The median gross composition of each beverage type expressed in % of total volume and the significance of contrasts between beverage groups. *P < 0.05, **P < 0.01, ***P < 0.001, ^†^*P* < 0.10. ns = not significant, na = analysis not applicable.

**Table 4:** Individual amino acids across beverages.

Trait	Rice	Soya	Coconut	Oat	Almond	Cow	Goat	Plants vs. animals	Fruits vs. crops	Monocots vs. dicots	Cereals vs. legumes
His	3.94	81.49	4.47	16.50	23.63	86.95	75.28	***	***	***	***
Arg	5.40	235.72	28.65	40.11	78.97	93.53	80.55	*	ns	***	***
Ser	4.87	163.09	11.70	35.89	31.73	159.31	151.06	***	ns	***	***
Gly	5.03	126.18	8.42	34.00	46.09	51.58	49.47	*	ns	***	***
Asp	8.15	416.91	19.45	68.52	96.46	255.37	246.44	***	ns	***	***
Glu	18.71	764.76	59.02	187.50	268.74	776.09	733.58	***	ns	***	***
Thr^a^	2.57	116.21	6.40	20.52	21.57	129.06	142.21	***	ns	***	***
Ala	2.73	132.82	11.70	30.75	35.84	100.39	101.32	***	ns	***	***
Pro	0.94	154.32	7.72	30.91	30.86	273.84	266.67	***	ns	***	***
Lys^a^	3.00	257.55	10.67	28.93	22.39	324.31	314.64	***	ns	***	***
Met^a^	0.50	20.92	2.63	6.34	3.22	58.79	45.49	***	*	***	***
Tyr	0.40	104.90	4.13	21.95	18.10	130.40	106.58	***	ns	***	***
Val^a^	1.70	118.64	8.75	25.30	29.21	168.98	166.07	***	ns	***	***
Cys	2.52	27.07	2.40	25.30	5.67	13.02	16.40	*	**	***	***
Ile^a^	1.60	120.33	3.38	17.23	22.33	136.91	126.38	***	ns	***	***
Leu^a^	4.20	219.10	12.10	44.20	51.77	278.32	265.43	***	ns	***	***
Phe^a^	2.48	78.98	5.54	31.82	18.20	6.38	6.29	^†^	ns	**	**
Trp^a^	1.60	45.88	2.06	11.18	7.16	48.11	46.86	***	*	***	***

The median amino acid content of each beverage type expressed in mg/100 g beverage and the significance of contrasts between beverage groups. *P < 0.05, **P < 0.01, ***P < 0.001, ^†^*P* < 0.10. ns = not significant, na = analysis not applicable.

^a^Essential amino acid.

However, considering the mere quantity of each amino acid may be misleading and should be done with caution. Indeed, a joint report by FAO and WHO^18^ drew global attention in 1991 to the quality of protein present in foodstuffs, hereunder referring specifically to the bioavailability of amino acids and the human gut’s ability to absorb said amino acids. They suggested the use of the so-called protein digestibility-corrected amino acid score (PDCAAS). Undoubtedly, the nature of proteins relates to a vast array of factors such as their tertiary structure, but also non-protein factors with which they may interact such as antinutrients, tannins, and fibre^18^. In particular, Kunitz trypsin inhibitors and Bowman-Birk inhibitors in soya require a heat treatment at very high temperatures for their deactivation, which is an additional processing step often implemented in commercial settings to optimise the nutritional benefit of the soya product at the expense of higher costs and resource-use^19,20^. What is more, the abundance of tannins in cereals can reduce protein digestibility^21^.

Based on the measurements of ileal protein matter in pigs, a quality scoring referred to as digestible indispensable amino acid score (DIAAS) was suggested in the 2011 FAO expert consultation report as the universal mean by which foodstuff protein quality should be evaluated, substituting the outdated PDCAAS^22^. It recognises the presence of each indispensable amino acid as an individual nutrient and evaluates its digestibility which varies among proteins^23^. The higher the score the greater the quality of the protein material in the food is said to be. In a recent study, soya, coconut, oat, rice, and almond-based beverages were given a score of 1.08, 0.72, 0.59, 0.43, and 0.39, respectively, when using an adult’s reference pattern. Cow milk, on the other hand, was given a DIAAS of 1.45. The older, but to date still more commonly referenced score in literature, PDCAAS, has also been widely applied to evaluate popular protein sources^18^. Similarly, cow’s milk has consistently scored a PDCAAS value of 1.00, soya 0.84–1.00, coconut 0.89–0.94, oat 0.54–0.57, almond 0.40, and rice 0.37–0.59^24–27^. Therefore, although almond-based beverages ranked the second PBB in terms of protein content, its poor DIAAS and PDCAAS scores may translate into consumers benefiting less from its protein than oat- or coconut-based beverages. It is thus evident that, with the generally low protein content and poor DIAAS and PDCAAS of PBB, these beverages do not represent substitutes for protein sources of animal milk. The exception is given by soya-based beverages, which beside having a greater protein content, also generally have high-quality protein, although the essential amino acid Met is the least abundant. It must be considered however, that 14% of people suffering from cow milk allergy, one of the main reasons for the PBB market growth, also experience allergies towards soya protein, and these beverages can thus not act as valid substitutes for cow milk^28^.

Although the protein intake is generally above adequate in the western world, certain demographic groups must be more attentive as their needs surpass the normal adult requirements. These include infants, children, and elderly consumers^10^. Not meeting the dietary requirements of essential amino acids could have negative physiological and biochemical consequences^29^. In particular, consumers following vegan diets should plan meticulously to meet said requirements as the poor DIAAS of plant products extends beyond beverages but also to any plant-based foodstuff. Indeed, animal-based protein sources are so well-adapt to human requirements in terms of essential amino acid composition that their ecological footprints, i.e. their land and water use, and their full life cycle CO_2_ emissions, are in general equal to, or lower than vegetal-based sources of proteins when considering amounts of foodstuff required to meet human requirement^30–32^ although soya-based beverages may be the exception^33^.

### Fat content and fatty acid profile

As expected, both cow and goat whole milk demonstrated the highest values of lipid content at 3.55% and 3.72%, respectively (Table 3), but also the lowest CV of 4% and 17%, respectively (Table 2). The plant group had statistically different lipid content from the animal group with the lowest found in oat-based beverages (0.37%) and the highest in almond-based beverages (1.99%). The plant-based lipids almost exclusively consisted of LCFA, aside from coconut which had high MCFA, while the animal milk consisted primarily of LCFA and MCFA (Table 5). In regard to the individual FA and their saturation groups, it is evident that a much larger fraction of the lipid content of animal milk comprised SFA compared to PBB, about 72 vs. 9.59–17.92% (Table 5). Again, the exception was given by coconut, which consisted of about 93% SFA. On the contrary, the USFA fraction was significantly larger in PBB than in animal milk, of which a significant difference within the PBB was evident between dicots (high) and monocots (low), probably due to the coconut-based beverages in the monocot group.

**Table 5:** Fatty acids across beverages.

Trait	Rice	Soya	Coconut	Oat	Almond	Cow	Goat	Plants vs. animals	Fruits vs. crops	Monocots vs. dicots	Cereals vs. legumes
*Individual*											
C4:0	0.00	0.00	0.00	0.00	0.00	1.96	1.66	***	ns	ns	na
C5:0	0.00	0.00	0.00	0.00	0.00	0.04	0.03	***	ns	ns	ns
C6:0	0.00	0.00	0.64	0.00	0.00	1.88	2.14	***	^†^	**	ns
C7:0	0.00	0.00	0.00	0.00	0.00	0.05	0.06	***	ns	ns	ns
C8:0	0.00	0.00	7.80	0.02	0.00	1.37	2.69	***	**	**	^†^
C9:0	0.00	0.00	0.00	0.00	0.00	0.07	0.10	***	na	na	na
C10:0	0.00	0.00	5.96	0.00	0.00	3.31	9.94	***	**	**	ns
C11:0	0.00	0.00	0.04	0.00	0.00	0.11	0.15	***	***	*	na
C12:0	0.00	0.00	47.01	0.00	0.00	3.93	5.28	***	**	**	^†^
C12:1	0.00	0.00	0.00	0.00	0.00	0.15	0.17	***	na	na	na
C13:0	0.00	0.00	0.05	0.00	0.00	0.16	0.12	***	***	**	ns
C13:1	0.00	0.00	0.00	0.00	0.00	0.14	0.10	***	na	na	na
C14.0	0.26	0.14	17.80	0.29	0.10	12.55	11.46	***	ns	***	***
C14.1	0.00	0.00	0.00	0.00	0.00	0.51	0.14	***	na	na	na
C15.0 iso	0.00	0.00	0.00	0.00	0.00	0.31	0.37	***	ns	ns	ns
C15.0 anti	0.00	0.00	0.00	0.00	0.00	0.51	0.36	***	na	na	na
C15.0	0.05	0.00	0.00	0.00	0.00	0.65	0.47	**	**	*	*
C16.0	8.51	10.80	9.14	14.78	7.04	34.53	27.75	***	***	^†^	ns
C16:1n7	0.18	0.12	0.00	0.17	0.54	0.94	0.49	*	ns	ns	*
C16:1n9	0.11	0.00	0.00	0.00	0.00	0.98	0.54	***	ns	ns	*
C17.0	0.06	0.12	0.00	0.05	0.03	0.60	0.56	***	***	**	***
C17.1	0.00	0.00	0.00	0.00	0.00	0.08	0.08	***	na	na	na
C18.0	2.77	4.35	3.12	2.33	1.99	9.40	8.58	***	*	ns	***
C18:1n7	0.88	1.27	0.06	0.90	1.24	1.93	1.59	***	ns	***	***
C18:1n9	45.74	22.42	5.26	38.84	62.10	19.53	19.10	**	ns	ns	***
C18:2n6^ac^	15.68	53.00	1.08	36.67	26.30	2.37	3.10	***	***	**	***
C18:3n3^ab^	0.37	7.38	0.00	0.67	0.03	0.49	0.65	ns	***	*	***
C18:3n6^c^ & C19:0	0.00	0.00	0.00	0.00	0.00	0.13	0.12	***	ns	ns	ns
C19:1	0.27	0.02	0.00	0.05	0.00	0.16	0.08	^†^	**	ns	*
C20:0	0.37	0.39	0.16	0.26	0.14	0.19	0.22	ns	***	ns	ns
C20:1	0.06	0.04	0.00	0.06	0.00	0.27	0.16	***	**	ns	ns
C20:1n9 & C20:1n7	0.44	0.22	0.00	0.73	0.00	0.00	0.00	***	***	ns	**
C20:2n6^c^	0.00	0.00	0.00	0.00	0.00	0.00	0.00	ns	*	*	ns
C20:3n6^c^ & C21	0.00	0.00	0.00	0.00	0.00	0.15	0.03	***	ns	ns	ns
C20.5n3^b^	0.00	0.00	0.00	0.12	0.00	0.05	0.03	**	ns	*	*
C20:4n6^c^	0.00	0.00	0.00	0.00	0.00	0.21	0.24	***	na	na	na
C22:0	0.62	0.36	0.00	0.26	0.11	0.11	0.11	**	***	ns	ns
C22:1n9	0.00	0.00	0.00	0.20	0.00	0.00	0.00	*	ns	*	**
C22:5n3^b^	0.00	0.00	0.00	0.00	0.00	0.08	0.18	***	ns	ns	ns
C22:6n3^b^	0.00	0.00	0.00	0.00	0.00	0.00	0.00	ns	^†^	^†^	ns
C24:0	0.40	0.01	0.00	0.26	0.00	0.00	0.00	***	***	**	***
*Group*											
SFA	14.19	15.78	93.57	17.92	9.59	72.10	73.34	***	ns	***	ns
MUFA	49.09	24.09	5.35	41.12	64.08	24.33	22.34	*	ns	ns	***
PUFA	16.14	60.81	1.08	37.83	26.41	3.30	4.41	***	***	***	***
USFA	85.81	84.22	6.43	82.08	90.42	27.71	26.49	***	ns	***	ns
SCFA	0.00	0.00	0.00	0.00	0.00	2.00	1.68	***	ns	ns	ns
MCFA	0.07	0.00	61.10	0.06	0.00	10.98	20.48	***	*	**	ns
LCFA	98.88	99.49	38.90	99.1	99.78	86.88	77.62	***	ns	***	***
VLCFA	1.05	0.44	0.00	0.78	0.16	0.19	0.29	*	***	*	***

The median fatty acid content of each beverage type expressed in g/100 g of total fatty acid, and the significance of contrasts between beverage groups. *P < 0.05, **P < 0.01, ***P < 0.001, ^†^*P* < 0.10, ns = not significant, na = na = analysis not applicable.

*SFA* saturated fatty acid, *MUFA* monounsaturated fatty acid, *PUFA* polyunsaturated fatty acid, *USFA* unsaturated fatty, *SCFA* short chain fatty acids, *MCFA* medium chain fatty acid, *LCFA* long chain fatty, *VLCFA* very long chain fatty.

^a^Essential fatty acid.

^b^Omega 3 family.

^c^Omega 6 family.

Indeed, the generally lower lipid content, and consequently lower caloric content, is one aspect that has drawn health-orientated consumers to PBB in the Western world, along with their very low levels of SFA^8^. In this case, mirroring the lipid content and profile of animal milk in PBB alternatives may not be desirable, at least not in the West. Partial or full skimmed animal milk remain options for consumers seeking products low in lipids and free from the negative health impacts of SFA.

Saturated fatty acids can be hypercholesteraemic causing increases in low-density lipoprotein (LDL) cholesterol^34,35^ and evidence suggest that their consumption increases the risk of developing cardiovascular diseases^29,36^ although this remains controversial^37^. This has led to dairy products becoming entangled in consumer beliefs of lowered health due to increased cardiovascular risk. Nonetheless, studies investigating dairy consumption and human cardiovascular health have found no negative correlations between the two^26,38^. Some studies even present evidence for reduced cardiovascular health risks with milk consumption^39^ which has been, in part, attributed to other substances found in dairy products offsetting the otherwise potentially harmful effects of elevated dietary SFA consumption^26^.

Nevertheless, the high USFA fraction, and particularly PUFA fraction in PBB, which, aside from coconut, was roughly 3 factors greater than in animal milk (Table 5), represents a great source of healthy lipids. Additionally, by adding external crop oils to PBB, these beneficial fatty acid fractions can be further elaborated, while simultaneously improving taste and mouthfeel^40^. Indeed, substituting SFA with PUFA have shown to reduce the level of LDL, while concurrently increasing high-density lipoprotein (HDL) cholesterol^35^. In fact, one of the two essential fatty acids, namely C18:2n6, constituted a significantly greater fraction in PBB lipids, hereunder particularly soya-based beverages and to a lesser extend oat-based beverages. This omega-6 fatty acid has been associated with reduced cardiovascular risk, improved long-term glycaemic control, and insulin resistance^41^. C18:2n6 also acts as a precursor to the pro-inflammatory FA C20:4n6 however, and it is therefore commonly believed that its intake promotes inflammation, although results are controversial^42^. The high content of C18:2n6 in the PBB may thus not be as beneficial to consumers as believed. On the other hand, the omega-3 essential fatty acid C18:3n3, which has been shown to possess neuroprotective properties^43^ was not statistically different between PBB and animal milk, but instead significantly different between groups of PBB, likely due to very high contents in soya-based beverages.

Furthermore, it is recognised that oat derivatives have additional positive health effects due to the abundance of soluble fibres and antioxidants capable of reducing triglycerides and LDL cholesterol^44^. Oat is rich in avenanthramides (about 300 mg/kg in kernels), phenolic compounds reported to exhibit antioxidant and anti-inflammatory activities^45–47^, while rice oil provide γ-oryzanol with similar effects^48^. However, the content of avenathramides is highly correlated with the whole grain oat content^49^. Since the actual oat content in the PBB ranged from just 10 to 16%, it is highly likely that the avenanthramide content is very limited, while the content of γ-oryzanol was not detectable in milled rice beverages in a recent study^50^.

Although the lipid content of milk provides essential caloric input important to many countries particularly in Africa, this focus has shifted in the western market where low-calorie, low-SFA products are sought after^51^. Coconut-based beverages frequently presented values of FA content dissimilar to other PBB (Table 5). This has been widely reported and discussed in literature^2,9,10^. Particularly, coconut-based beverages consisted of a much larger SFA and MCFA fraction than its plant counterparts, while simultaneously exhibiting smaller fractions of USFA, LCFA, and VLCFA (Table 5). On these grounds, coconut-based beverages appear to approximate the lipid composition of animal milk.

Although this could be interpreted as coconut-based beverages being less healthy than other PBB, one must look at the more detailed composition of the FA, and indeed, links between coconut oil consumption and cardiovascular diseases remain scarce and highly controversial^52^. The detailed lipid composition of the coconut-based PBB highlight C12:0 (47% of the total) and C14:0 (17.8% of the total) as the most abundant FA, both of which have been reported to lower LDL content^53^, while C12:0 has also been reported to possess anti-cancerous and antiviral effects^52^. Medium chain fatty acids have been shown to favour weight control as they are preferentially oxidised and transported to the liver similarly as carbohydrates, to be used as a rapid source of energy. This is contrary to LCFA which, due to their large size, preferentially become stored in the adipose tissue unless their energy is required immediately^54,55^. Therefore, although the SFA content of coconut-based beverages is higher than that of animal milk, the SFA profile in coconut-based beverages advantageously differ from those in animal milk.

### Total carbohydrates, sugar, and additives

Rice-based beverages contained the greatest amount of carbohydrates (Table 3), which was also reflected in their high dry matter (DM) (12.31%). Although glucose content was the highest in rice-based beverages (3.12%), this high carbohydrate content was probably due to the extremely high starch content in rice, similarly as to the other cereal-based beverage oat. The main energy storage form in cereals is starch, constituting up to 60–75% of the grain’s weight^56^, indicating that consumers wishing a low-carb diet should avoid cereal-based beverages. Oat-based beverages indeed contained the third-most carbohydrate (8%), immediately after almond (8.2%). The high carbohydrate content of the almond-based beverages was unexpected as the innate carbohydrate content of the almond nut is very low. However, this may be due to two coconut brands containing the polysaccharide xanthan gum and carbohydrate-binding sunflower lectin (Table 1), thereby inflating the total carbohydrate content. Indeed, almond had the highest CV for carbohydrate content (77%; Table 2). As expected, only cow and goat milk contained lactose (4.72% and 4.31%, respectively). Glucose and fructose content were significantly higher in PBB with almond-based beverages containing the most fructose (Table 3). The difference between PBB groups was more statistically significant for fructose than for glucose, likely due to exploitation of the higher sweetening power of fructose in naturally less sweet products. The difference between monocots vs. dicots and cereal vs. legume was highly significant in fructose, but only moderately significant in glucose (Table 3). Glucose was not significantly different between the fruit and crop group, but fructose was weakly significant (Table 3). Taste, colour, and consistency of PBB are often improved by the use of additives such as sucrose to fit consumer expectations of milk alternatives^7^. Indeed, 73% of the tested PBB brands contained added salt, 23% of them added sugar, and 67% contained at least one additive other than salt, sugar, and water (e.g. xanthum gum, sunflower oil, aromas; Table 1). With addition of sugar, the glycaemic-index of the PBB is increased, which impairs the consumer’s ability to control blood glucose levels, similarly with products containing high levels of starch, which the rice and oat-based beverages appear to do. A low glycaemic load diet has been linked to reduced risks of cardiovascular disease, obesity, and diabetes^28^. Furthermore, high sodium diets are known to cause increased blood pressure and risk of kidney diseases^57^ and the addition of salt may therefore also impair the nutritional quality of the PBB.

The perceived advantageous health effects of PBB in terms of low SFA content and increased PUFA fractions may thus be counteracted by the use of these additives and the high carbohydrate content in certain PBB groups (cereals as well as in almond). In fact, studies suggest that substituting SFA with carbohydrate, and in particular highly refined carbohydrates such as sugar, results in elevated levels of triglycerides and LDL particles while concurrently reducing HDL cholesterol^54^. Such effects are of particular concern in Western settings where the prevalence of obesity and insulin resistance is an ever-growing problem.

Moreover, PBB are often referred to as “highly processed” products with oat-based beverages requiring on average 14 production steps, almond 15, and soya 13. These processes involve steps such as soaking, grinding, blanching, cooking, and filtering^31^. This does not include steps for improving the nutritive profile of said PBB. In comparison, fresh milk requires only two major steps: pasteurisation and homogenisation^31^. These additional processing steps add to the PBBs’ ecological footprint^33^, which due to their poor nutritional quality, has been estimated to be equal to or greater than those of dairy products’ ecological footprint considering their nutrient density and not kg^32^. It must therefore also be carefully considered if the PBB really is a low-impact alternative to the sustainable-oriented consumer.

### Minerals

Iodine was exclusively found in cow and goat milk (242 and 377 µg/kg; Table 6). This mirrors data obtained by other groups in similar studies, undergone in other countries. Walther et al. analysed a set of PBB, taken from the Swiss market and found significant I content only in the subset of PBB to which red algae had been added, an additive used as a source of I fortification^10^. As opposed to Walther et al., fortified products were excluded from the current study, and no product containing a significant content of I was thus obtained. Escobar et al. carried out a descriptive study, analysing the composition of 179 commercial PBB available on the Spanish market, as declared from labelling. No I-fortified PBB are available on the Spanish market, thus none of the products analysed reported a significant I content^58^. Since dairy products have a central role in contributing to I adequacy in many countries^14,59^, the progressive reduction of their consumption and the shift towards a more plant-based diet risks to affect I adequacy. Indeed, increases in mild I-deficiencies currently observed in Australia and continental Europe has been linked to the reduction in the use of sanitising iodophors in the dairy industry which leave I residues in milk^60^. This demonstrates the importance of dairy products in meeting I requirements in these geographical areas. Therefore, to cover the recommended intakes, especially during infancy, pregnancy, and breastfeeding, the industry should ensure efforts are made to promote I fortification in PBB.

**Table 6:** Minerals content across beverages.

Trait	Rice	Soya	Coconut	Oat	Almond	Cow	Goat	Plants vs. animals	Fruits vs. crops	Monocots vs. dicots	Cereals vs. legumes
I	0	0	0	0	0	242	377	***	^†^	ns	na
Ca	125	260	133	139	214	1067	882	***	ns	***	***
P	84	471	58	138	131	930	1000	***	***	***	***
Mg	0	184	15	9	65	88	117	ns	ns	*	ns
K	0	1394	337	323	218	1408	1636	***	ns	**	***
Na	228	325	367	381	253	405	878	***	ns	ns	ns
S	0	212	0	5	0	241	234	***	***	***	***

The median mineral content of each beverage type expressed in mg/kg beverage, except for iodine which is expressed in µg/kg beverage, and the significance of contrasts between beverage groups. *P < 0.05, **P < 0.01, ***P < 0.001, ^†^*P* < 0.10, ns = not significant, na = analysis not applicable.

All minerals were in general present in higher concentrations in animal milk than PBB, except for Mg whose content was statistically similar between animal milk and PBB (Table 6). Rice-based beverages were the most mineral-poor beverages, lacking Mg, K, and S and demonstrating low to moderate contents of Ca, P, and Na (124, 84 and 228 mg/kg, respectively). This is also mirrored in rice-beverages low ash content at 0.09% (Table 3). On the contrary, soya-based beverages demonstrated the greatest mineral content among the PBB, which is further evident in its high ash content (0.45%). Na was the only mineral found in greater amounts in other PBB than soya (325 mg/kg), namely in coconut- and oat-based beverages (367 and 381 mg/kg; Table 6). This could be due to salt being added in higher concentrations in coconut- and oat-based beverages than in soya (Table 1). Calcium content was about 10-times greater in cow milk than in the PBB (about 1000 mg/L), with soya-based beverages demonstrating the largest calcium content of the PBB (260 mg/L). Legumes, whole-grains, and nuts are known to be great sources of certain minerals, i.e. K, P, and Mg, although this is not reflected in the mineral content of the PBB, suggesting that flours of dehulled cereal grains were probably used for their preparation. This low mineral content could also be explained by the plant material constituting a small percentage of the total volume of the PBB. Indeed, the content of the main plant origin of each PBB ranged from just 3% in one almond brand to 40% in one coconut brand (Table 1).

Due to the important role Ca plays in bone development^31^, milk has been campaigned as a great nutritive source for healthy bones, particularly in growing children. For this reason, many PBB products are fortified with Ca to mirror concentrations found in cow milk. According to the EFSA (2017)^61^, an adult over the age of 25 requires 950 mg of Ca per day, which can be met with 890 mL of cow milk or 1077 mL goat milk. On the contrary, despite being the PBB with the highest Ca content, 3654 mL of unfortified soya-based beverage are needed to obtain the required daily intake of Ca. Similarly, 330 mL of cow milk will provide five-times the required daily P intake, and 13% and 22% of the daily requirements of K and Mg, respectively, while with 330 mL soya-based beverage these values are 28%, 13%, and 17%^61^. As for the poorest of the PBB in terms of mineral content, namely rice-based beverages, 7600 mL of beverage are required to obtain the 950 mg Ca requirement, while 330 mL will not provide any substantial amount of neither P, K, nor Mg^61^.

### Overall nutritional value

Beyond the mere content of individual macro and micronutrients, one must always consider the overall content of each beverage and their bioavailability. The DM confers information on percentage of solids present in foodstuff, and therefore indirectly on amount of total nutrients. Rice-based beverages were found to have the highest DM at 12.31%, followed by cow and goat milk at 12.22% and 11.84% (Table 3), respectively. However, these high DM values constitute highly diverse nutrient profiles. As regards rice, carbohydrates represented the largest fraction of the DM at 12.60%, most of which was likely starch^56^. On the other hand, the DM of animal milk constituted in large parts of carbohydrate, hereunder mainly lactose, as well as lipids and protein. The generally high carbohydrate content in PBB, except for coconut and soya, with concurrently low mineral, protein, and lipid contents make these products poor sources of nutrients in relation to the energy provided. Although the low SFA and high PUFA contents in PBB may represent healthy FA profiles associated with reduced risk of cardiovascular disease onset, the amount of FA per 100 mL is still very limited. What is more, the lack of evidence on the presence of the often-referenced health-promoting avenanthramides and γ-oryzanol after oat and rice PBB processing, does not strengthen the nutritional profile of these two PBB^49,50^.

Milk therefore represents a matrix with a more nutritive profile than PBB, with the exception given by soya. Soya-based beverages sustain similar albeit lower mineral contents in comparison to milk, however, and can therefore not act as a complete substitute. This is of particular concern to I, Ca, and P. However, even mineral fortification may not be a mean by which to overcome this issue. Craig and Fresán^62^ found that the amount of Ca absorbed from Ca_3_(PO_4_)_2_, a common compound used as a Ca fortifier, was significantly less than Ca absorbed from milk. Concurrently, the amount absorbed from CaCO_3_, another popular Ca fortifying compound, was comparable to that of milk^62^. However, issues regarding sedimentation of these Ca fortifiers, which occurs during the product’s shelf life, persist^31^.

Research into the bioavailability of minerals in the matrices of PBB is still lacking. Nonetheless, it is evident that some phyto-compounds actively block the absorption of certain minerals exacerbating the issue of low mineral contents in PBB. Phytates, used by plants as storage compounds for P and inositol, are grouped as so-called antinutrients. They are found in cereals, nuts, and seeds, with particularly oat having high concentrations at 2618 mg/100 g dry matter^63^. They form soluble complexes with divalent cations under acidic environments, like those found in the digestive tract. Phytates can thus sequester dietary Z, Fe, and Ca, and since monogastrics such as humans do not produce the phytase enzyme^64^, the minerals remain unavailable for absorption^65^. Phytates are heat stable^66^ and are therefore not removed or denatured during the common thermal processing of PBB. Raghavendra et al. explored their content in soya-based beverages and found it to be 0.106 g/100 g beverage. With fermentation using the phytate-degrading *Pediococcus pentosaceus*, the concentration of phytates decreased to 0.093 g/100 g, a reduction of 12%, and the Ca bioavailability concurrently increased by 68%^67^. This, however, required a 12 h fermentation step not compatible with commercial production.

Thus, although PBB may be marketed as nutritive dairy alternatives low in cholesterol and cardiovascular disease-inducing FA, their nutrient content is highly limited, and their carbohydrate and sugar contents elevated. Due to the presence of anti-nutritive phyto-compunds such as phytates, and their low endogenous mineral content and high carbohydrate content, if implemented in the diet to fully substitute dairy products, issues regarding mineral deficiencies and blood glycaemic control may arise. This is of special concern for vulnerable demographic groups such as infants, elderly, and lactating women if implemented without regards to nutritional requirements as may be the case if proper information is not correctly communicated. Indeed, the high CV of most traits in comparison to milk demonstrates the lack of standardisation across PBB manufacturing, making informed decision-making by consumers difficult.

### Discriminant ability of composition traits

**Fig. 1:**
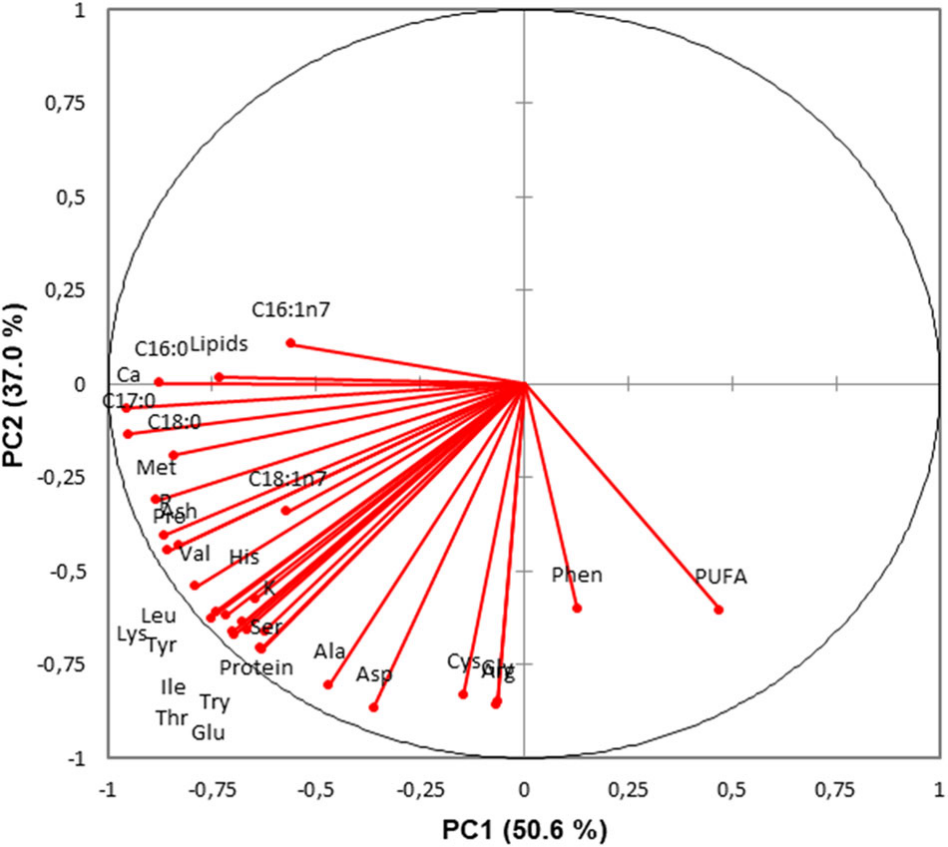
The contribution of each trait to the first two principal components (PC). Vectors depict the loadings of each trait; traits exclusively with loadings >0.5 are included.

**Fig. 2:**
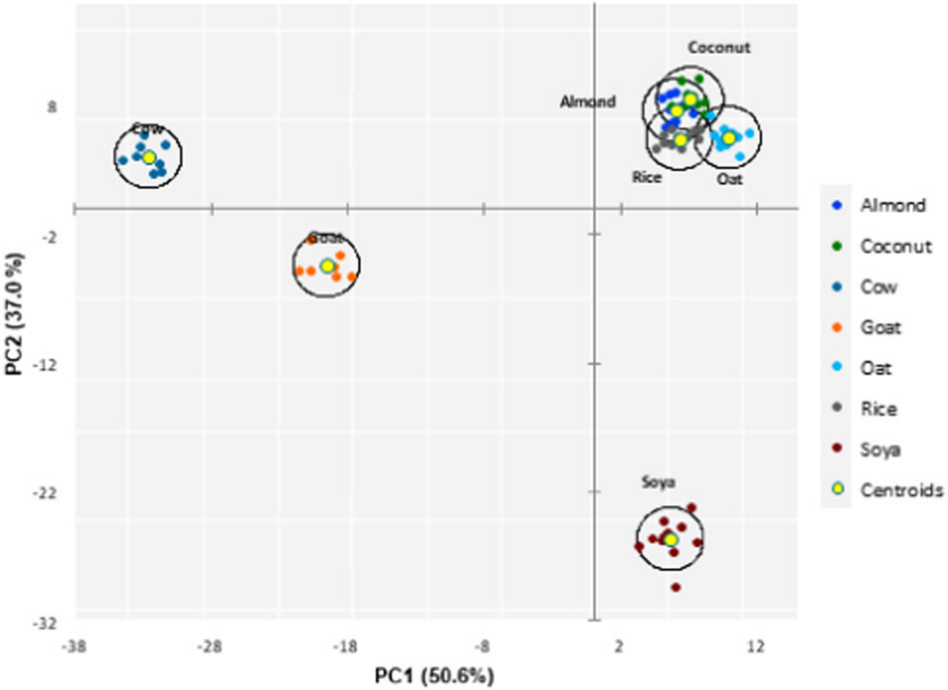
2D-plot of the unsupervised approach. Principal component analysis, where PC stands for principal component whose % variance explained is given in parenthesis.

**Fig. 3:**
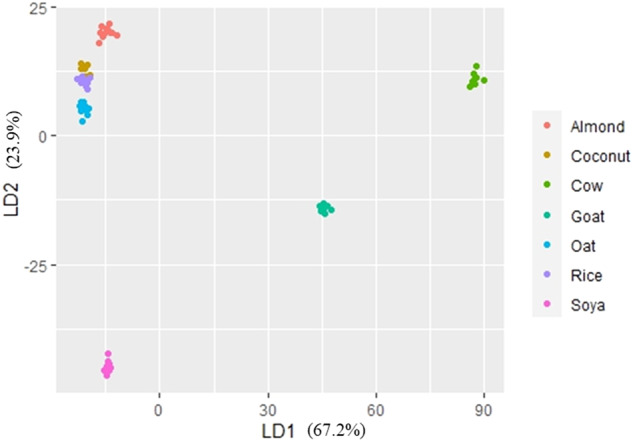
2-D plot of the supervised approach. Linear discriminant analysis, where LD stands for linear discriminant function whose % variance explained is given in parenthesis.

The PCA on the common traits among all beverage types revealed PC1 and PC2 to explain 50.6% and 37.0% of the variance, respectively (Figs. 1 and 2). The five most influential traits of each principal component included Leu, Ser, Val, Pro, and Met for PC1, and Glu, Asp, Tyr, Leu, and Lys for PC2 (Fig. 1; Table 7). The beverages formed clear clusters when plotted on a 2D-plot, with particularly cow and goat milk clearly segregating from the PBB (Fig. 2). The soya-based beverages were the PBB most dissimilar from the other PBB, while almond, oat, coconut, and rice-based beverages formed an overlapping cluster (Fig. 2). The PCA therefore demonstrates that the nutrient composition is indeed different not just between the animal and PBB but also between soya-based beverages and all other PBB, and that despite the high variability between brands of PBB, there is a somewhat accordance between them. This corroborates the fact that PBB differ according to the manufacturing process, recipe, and basic material. The supervised LD analysis (Fig. 3; Table 7) showed excellent discrimination ability; 100% of the beverages were correctly classified, with 14.9% being of rice and coconut origin each, 16.2% of soya, oat, and almond origin each, and 10.8% of cow and goat origin each. Indeed, with LD1 and LD2, the LD analysis was able to maximise the class separability, explaining 67.2% and 23.9% of the variance, respectively (Fig. 3). This study used detailed FA and amino acid composition, as well as mineral content and gross composition to identify the best discriminatory traits between different PBB and milk. These were determined as the different FA saturation groups, and the two individual FA C18:1n9 and C16:0, as well as Glu (Table 7). The differing contents of the various FA groups is indeed one of the main factors driving health-orientated consumers from animal milk to PBB, in which it is evident that even within PBB, differences exist (Table 5; Fig. 3). Consumers must therefore consider which type of PBB best accommodates their dietary aspirations and needs. Analyses highlight that the almond-, coconut-, rice- and, oat-based PBB are similar in nutritional content, while soya-based PBB differ highly from other PBB. The LD demonstrated that cow milk is more similar to PBB other than soya in regard to LD2, while goat milk is more similar to PBB in regard to LD1.

**Table 7:** The top discriminatory traits identified in the principal component analysis and the linear discriminant analysis.

Principal component analysis		Linear discriminant analysis	
Trait	Coefficient	Trait	Coefficient
PC 1		LD1	
Leu	−10.2	USFA	2845.5
Ser	6.0	PUFA	−1984.6
Val	5.6	MUFA	−1874.3
Pro	5.1	C18:1n9	−225.7
Met	−3.4	SFA	−172.8
PC 2		LD2	
Glu	6.9	USFA	127.9
Asp	−6.6	MUFA	−100.0
Tyr	4.3	PUFA	−77.7
Leu	−2.4	Glu	22.4
Lys	−2.2	C16:0	−15.3

*PC* principal component, *LD* linear discriminant function, *SFA* saturated fatty acid, *MUFA* monounsaturated fatty acid, *PUFA* polyunsaturated fatty acid, *USFA* unsaturated fatty acid.

It can thus be concluded that the nutrition profile of the investigated PBB is not of comparative quality to those of both cow and goat milk, with the exception given by soya. The I, Ca, and P content, however, remained greatly elevated in milk in comparison to the soya-based beverages, while also the FA profile differed. Oat, rice, almond, and coconut-based beverages all had significantly lower protein and essential amino acid content than animal milk. Soya, however, had consistently comparable content of protein and essential amino acids to animal milk, but due to lower bioavailability and poorer DIAAS, consumers may not benefit from these nutrients to the same extended were they sourced from animal milk. Coconut FA composition mirrored to a certain extent that of the two milk types, but the USFA present in coconut-based beverages are considered to possess beneficial health effects and should therefore not be considered a negative aspect of this PBB. Both cow and goat milk represent great sources of most minerals, unlike PBB which were generally mineral-poor and implicated in issues regarding bioavailability due to endogenous antinutrients. The variability between brands of the same PBB was extremely high, unlike for animal milk. This reflects a necessity for industry standardisation concerning PBB production so to gain consumer assurance. For this reason, further analysis with more PBB brands is required to fully elucidate the nutrient profile of PBB currently available on the market. What is more, although milk is not regarded as a vital source of vitamins, they remain a highly important part of the human diet and should also be taken into consideration in future studies. The combination of poor nutritional profile and need for a high level of processing nullifies what consumers often believe to be a nutritious alternative to traditional animal milk.

## Methods

### Samples

A total of 60 PBB, 8 UHT whole cow milk products, and 8 UHT whole goat milk products were purchased around Vicenza province (Northern Italy) and analysed between February and March 2022. The PBB belonged to different commercial brands (Table 1) and 12 samples were available for rice, oat, soya, almond, and coconut-based beverages each. One sample of rice and one sample of coconut-based beverages were later removed due to incomplete measurements and were not considered in any analyses. The chosen milk products also originated from different manufacturing plants (Table 1). To allow for a fair comparison of the nutrient composition between the different beverages, only products without mineral or vitamin fortification were chosen. The addition of sugars and salt is common practice during PBB processing to increase palatability. To have representative samples of products available on the market, some PBB containing added sugars and salt as declared on the label were also analysed (Table 1). Samples were kept at room temperature before analysis. To allow statistical comparison of the different beverage types and to elucidate tendencies within categories of PBB, groups of products representing different forms or origins of beverages were thereafter created as following:

- animal (cow and goat milk);
- PBB (rice, oat, soya, almond, and coconut);
- crop; includes plants grown in agricultural settings other than plantations (rice, oat, and soya);
- legume; includes pod fruits from the leguminous-family (soya);
- cereal; includes plants commonly referred to as grains (rice and oat);
- fruit; includes plants commonly grown in orchards (almond and coconut);
- monocot species (rice, oat, and coconut);
- dicot species (soya and almond).

Monocot and dicot refer to the two fundamental botanical classes of flowering plants which greatly differ in growth and developmental patterns and therefore also morphology^68^. The main difference refers to the presence of one or two cotyledons in their seeds/fruits used for the preparation of PBB.

### Analysis of composition

Each sample was analysed for dry mass (%), gross composition (%), FA profile (g/100 g), and amino acid (mg/100 g beverage), mineral (mg/kg beverage), and I (µg/kg beverage) content in certified laboratories.

#### Gross Composition

Samples were initially freeze-dried so that the mass of the sample thereafter represented only the DM percentage.

To determine the ash content, 5 g of DM sample was calcined in a muffle oven at 550 °C following AOAC 17th ED. 2000; method 945.46^69^.

Crude protein was determined according to the Kjeldhal method following AOAC 17th ED. 2000; method 991.20^70^. Briefly, the DM samples were mineralised in sulfuric acid catalyst. The resulting N-NH_3_ produced was distilled into a solution that was then titrated (Kjeltec 8400 FOSS TECATOR automatic titrator).

For the determination of crude lipid content, DM samples were initially hydrolysed using 4 M HCl whereafter petroleum ether:diethyl ether (50:50 v/v) was added to obtain the ether extract using a SOXTET 255 FOSS TECTOR extractor.

Fructose and glucose were extracted by the use of a 0.1 N sulfuric acid solution, while lactose was extracted using Carrez I (K_4_[Fe(CN)_6_]x 3H_2_O) and Carrez II (ZnSO_4_x7H_2_O) salts. Fructose, glucose, and lactose contents were subsequently determined via high-performance liquid chromatography (HPLC; Jasco Corporation, Tokyo, Japan). The HPLC system, consisting of an Aminex HPX 87H ion exclusion column (300 mm × 7.8 mm, Biorad) was equipped with a Jasco CO-2060 column oven (65 °C) and a Jasco RI-2031 Plus Refractive Index spectrometer. Diluted sulfuric acid (0.0025 N) was used as the mobile phase with an isocratic flow of 0.6 mL/min. ChromNav software was used for data acquisition.

Total carbohydrate content was not directly quantified but was retrieved from the declared nutrient contents on the packaging of each brand.

#### Amino acids

His, Arg, Ser, Gly, Asp, Glu, Thr, Ala, Pro, Lys, Met, Tyr, Val, Cys, Iso, Leu, Phe, and Trp were determined in both animal milk and PBB. Amino acids were analysed after hydrolysis specific to the type of amino acids and after pre-column derivatisation with 6-aminoquinolyl-N-hydroxysuccinimidyl carbamate. Their separation and quantification were performed through the use of an Agilent 1260 Infinity HPLC (Agilent Technologies, Santa Clara, CA, USA) with a reversed-phase C18 column (CORTECS C18, 2.7 µm, 2.1 × 150 mm) and a diode detector (Diode array Detector Agilent 1260 Series, DAD VL+) installed. The temperature was maintained at 45 °C. The determination of all the amino acids was carried out following the protein hydrolysis reference Method 1, Method 5, and Method 7 available in European Pharmacopoeia (2003) and included hydrolysis at 105 °C for 24 h with 6 M hydrochloride acid. Neutralisation with sodium hydroxide (8 M) occurred before volume adjustment and filtration (0.45 µm) and finally derivatisation was performed with the AccQ-Tag Ultra Derivatization Kit following manufacturer’s instructions (Waters Corporation, Milford, MA, USA). For Trp determination, the method described in the European Commission Directive 2000/45/EC (2000) was adapted and included basic hydrolysis with barium hydroxide (105 °C for 24 h)^71^.

#### Fatty acids

FA were extracted from the samples by adding hexane:isopropanol (3:2 v/v) at 110 °C for three extraction cycles, with a static period of 2 min, a flush volume 100%, and a purge of 60 s. The weight of each sample was noted after having dried under nitrogen flow, heat treated at 60 °C until dry, and lastly cooled in a desiccator. For FA determination, esterification using 2 mL methanol containing 1% v/v H_2_SO_4_ was conducted overnight at 50 °C. Phase shift was thereafter induced by addition of a hexane solution with 0.47 M Na_2_SO_4_ and the hexane phase was collected and quantified in the gas chromatograph (GC Agilent 7820) equipped with a G4567A automatic ampler (Agilent Technologies, Santa Clara, CA, USA) and a flame ionisation detector. The capillary column (30 m × 0.25 mm, with a film thickness of 0.25 µm) comprised an Omegawax capillary GC column (24136 Supelco; Sigma-Aldrich, Castle Hill, Australia). Hydrogen was used as the carrier gas (flow rate: 1.42 mL/min, 39.5 cm/s). The initial oven temperature was set at 50 °C and held for 2 min, whereafter it increased by 4 °C/min until reaching 220 °C where it was maintained for 18 min. The individual FA were identified by comparing their retention times with those of a standard (Supelco FAME mixC4-C24 #18919-1AMP; Sigma-Aldrich), however, due to the overlap of some FA on the chromatogram, not all FA could not be distinguished and thus correctly quantified, hereunder also some omega-3 and omega-6 FA. Peak areas were calculated using GC/MSD ChemStation software (Agilent Technologies) and expressed as a percentage of FA.

For the purpose of this study, fatty acids were grouped according to their saturation and chain length as follows:

- Saturated fatty acid (SFA): C4:0, C5:0, C6:0, C7:0, C8:0, C9:0, C10:0, C12:0, C13:0, C14:0, C15:0 iso & anti, C15:0, C16:0, C17:0, C18:0, C20:0, C22:0, C24:0.
- Monounsaturated fatty acid (MUFA): C12:1, C13:1, C14:1, C16:1n9, C16:1n7, C17:1, C18:1n9, C19:1, C20:1, C20:1n9 & C20:1n7, C22:1n9.
- Polyunsaturated fatty acid (PUFA): C18:2n6, C18:3n3, C20:2n6, C20:4n6.
- Unsaturated fatty acid (USFA): C14:1, C16:1n9, C16:1n7, C18:1n9, C18:2n6, C18:3n3, C19:1, C20:1, C20:1n9 & C20:1n7, C20:4n6, C20:2n6, C22:1n9.
- Short chain fatty acids (SCFA): C4, C5.
- Medium chain fatty acid (MCFA): C6:0, C7:0, C8:0, C9:0, C10:0, C11:0.
- Long chain fatty acid (LCFA): C12:0, C12:1, C13:0, C13:1, C14:0, C14:1, C15:0 iso, C15:0 anti, C15:0, C16:0, C16:1n9, C16:1n7, C17:1, C18:0, C18:1n7, C18:1n9, C18:2n6, C18:3n3, C18:3n6, C19:0, C19:1.
- Very long chain fatty acid (VLCFA): C20:0, C20:1, C20:1n9, C20:1n7, C20:2n6, C20:3n6, C20:4n6, C20:5n3, C21:0, C22:0, C22:1n9, C22:5n3, CC22:6n3, C24:0.

#### Minerals

The concentrations of Ca, Cr, Fe, P, Mg, Pb, K, Cu, Se, Na, Zn, S, and I were quantified. Samples were firstly mineralised to release inorganic minerals via microwave acid-digestion using the MILESTONE START D (Milestone Srl Sorisole BERGAMO) 1200 W power instrument equipped with a SK-10 high-pressure ROTOR (100 bar) and under thermal control by a temperature probe. The added reagents included super-pure 67% nitric acid and 30% hydrogen peroxide v/v. Samples were brought to 200 °C over 15 min in the temperature ramp phase and the temperature was maintained for 18 min. The samples were thereafter cooled to 35 °C. The mineralised samples were dissolved in demineralised water and quantified using inductively coupled plasma optical emission spectroscopy (ICP-OES) SPECTRO ARCOS (SPECTRO Analytical Instruments GmbH, Kleve, Germany). The method for the determination of minerals followed the method described in Poitevin^72^. The accuracy and precision of the method was evaluated by analysing the blank solution, the low-level control solution (recovery limits ± 30%) and the medium-level control solution (recovery limits ± 10%). The measured values were in excellent agreement for all minerals. Concentrations of Cr, Fe, Pb, Cu, Se, Zn, and S were below detectable levels for all PBB and milk brands and were therefore not considered in further analyses.

In the case of I, samples underwent a preliminary mineralisation. As such, 1 mL of the sample was diluted in 24 mL of an ammonia water solution (0.6%). Following gentle agitation, the samples were placed in a 90 °C water bath for 60 min to thereafter be cooled to room temperature and filtered using 0.45 µm syringe filter. A sample of 5 mL of the filtrate was diluted in ammonia solution (0.6% v/v) and quantified by inductively coupled plasma mass spectrometry (ICP-MS)^73^.

### Statistical analysis

The R software (v. 4.2.2, R Core Team, 2002)^74^ was used for data visualisation and analysis. Due to the non-normal distribution of the data for most of the variables, the comparison relied on the medians of different groups which were tested through the Mann–Whitney U test. The coefficient of variation (CV, %) was calculated for all traits within beverage type using Eq. (1) and presented in Table 2.

1 $${{\rm{CV}}}\left( \% \right)=100\times \frac{{\rm{Standard}}\; {\rm{deviation}}}{{\rm{Mean}}}$$

To understand which composition traits explained most of the observed variability of the different types of beverages, both an unsupervised and a supervised approach were used: a principal component analysis (PCA) and a linear discriminant (LD) analysis. The data was standardised by means of *z-*score normalisation, i.e., by subtracting the mean and dividing by the standard deviation within each variable. Furthermore, only traits with a lodging > 0.5 were included in the PCA. The software XL-STAT (Addinsoftware, France) for Microsoft Excel as well as the FactoMineR package^75^ and the MASS package^76^ available for the R software were used.

### Reporting summary

Further information on research design is available in the Nature Research Reporting Summary linked to this article.

### Supplementary information

Reporting summary

## Data Availability

The data that supports the findings of this study are available in Havard Dataverse with the identifier 10.7910/DVN/U9PNZH.
